# Understanding Ligand Binding to G-Protein Coupled Receptors Using Multiscale Simulations

**DOI:** 10.3389/fmolb.2019.00029

**Published:** 2019-05-03

**Authors:** Mercedes Alfonso-Prieto, Luciano Navarini, Paolo Carloni

**Affiliations:** ^1^Institute for Advanced Simulation IAS-5 and Institute of Neuroscience and Medicine INM-9, Computational Biomedicine, Forschungszentrum Jülich, Jülich, Germany; ^2^Medical Faculty, Cécile and Oskar Vogt Institute for Brain Research, Heinrich Heine University Düsseldorf, Düsseldorf, Germany; ^3^illycafè S.p.A, Trieste, Italy; ^4^Institute for Neuroscience and Medicine INM-11, Forschungszentrum Jülich, Jülich, Germany; ^5^Department of Physics, Rheinisch-Westfälische Technische Hochschule (RWTH) Aachen University, Aachen, Germany; ^6^VNU Key Laboratory “Multiscale Simulation of Complex Systems”, VNU University of Science, Vietnam National University, Hanoi, Vietnam

**Keywords:** G-protein coupled receptor, molecular dynamics, multiscale simulations, molecular mechanics, coarse grained, chemosensory receptors, bitter taste receptors, olfactory receptors

## Abstract

Human G-protein coupled receptors (GPCRs) convey a wide variety of extracellular signals inside the cell and they are one of the main targets for pharmaceutical intervention. Rational drug design requires structural information on these receptors; however, the number of experimental structures is scarce. This gap can be filled by computational models, based on homology modeling and docking techniques. Nonetheless, the low sequence identity across GPCRs and the chemical diversity of their ligands may limit the quality of these models and hence refinement using molecular dynamics simulations is recommended. This is the case for olfactory and bitter taste receptors, which constitute the first and third largest GPCR groups and show sequence identities with the available GPCR templates below 20%. We have developed a molecular dynamics approach, based on the combination of molecular mechanics and coarse grained (MM/CG), tailored to study ligand binding in GPCRs. This approach has been applied so far to bitter taste receptor complexes, showing significant predictive power. The protein/ligand interactions observed in the simulations were consistent with extensive mutagenesis and functional data. Moreover, the simulations predicted several binding residues not previously tested, which were subsequently verified by carrying out additional experiments. Comparison of the simulations of two bitter taste receptors with different ligand selectivity also provided some insights into the binding determinants of bitter taste receptors. Although the MM/CG approach has been applied so far to a limited number of GPCR/ligand complexes, the excellent agreement of the computational models with the mutagenesis and functional data supports the applicability of this method to other GPCRs for which experimental structures are missing. This is particularly important for the challenging case of GPCRs with low sequence identity with available templates, for which molecular docking shows limited predictive power.

## Introduction

G-protein coupled receptors (GPCRs) are one of the largest protein superfamilies, with more than 800 (4%) genes in humans (Venter et al., 2001; Fredriksson et al., 2003; Lagerstrom and Schioth, 2008; Tikhonova and Fourmy, 2010). They detect a wide variety of extracellular signals (from photons to hormones and neurotransmitters) and trigger a myriad of intracellular transduction cascades (using different G-proteins and second messengers) (Alexander et al., 2017). These pleiotropic receptors are involved in many physiological functions, from vision to chemical sensing and neurotransmission, and, hence, they are attractive targets for pharmaceutical intervention. Approximately 34% of currently FDA-approved drugs bind to GPCRs (Hauser et al., 2018) and they are used to treat disorders as diverse as pain, hypertension, diabetes, cancer or neurological diseases (Hauser et al., 2017). Given the physiological and pharmacological relevance of GPCRs, unraveling their ligand binding determinants can be extremely useful both for understanding receptor function and for designing new drugs.

**Figure 1:**
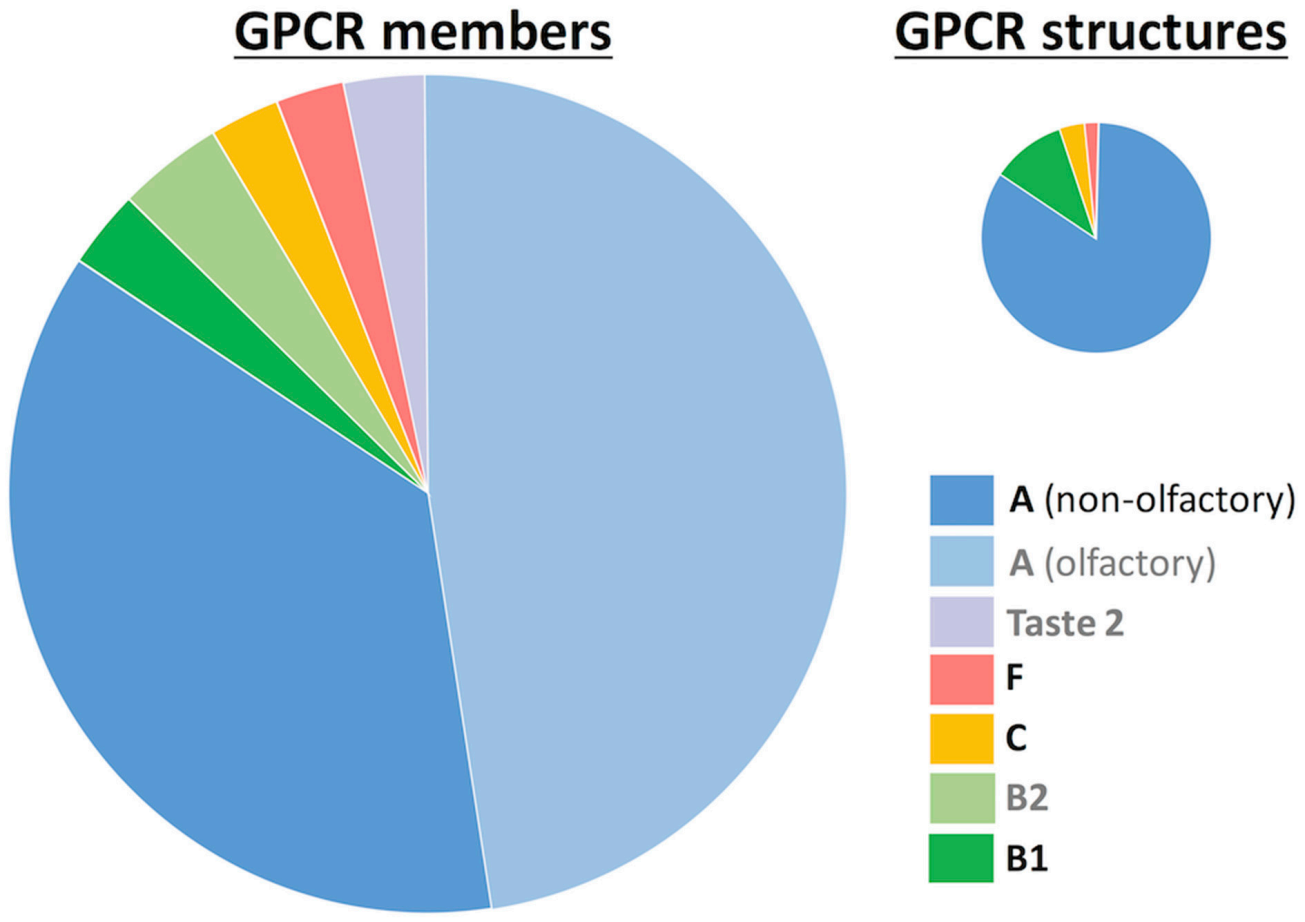
GPCR statistics. The number of members is based on reference (Munk et al., 2016a) and the number of experimental structures was taken from the GPCRdb database (https://gpcrdb.org/structure/statistics, accessed on January 2019). GPCRs are grouped according to the class A-F nomenclature (Fredriksson et al., 2003). Within class A, two groups are differentiated: non-olfactory and olfactory receptors. The taste 2 receptors have been proposed to belong to either class A (Nordstrom et al., 2011) or class F (Fredriksson et al., 2003), or even constitute a novel, sixth class (Munk et al., 2016a). Legend labels for those groups without experimental structures are in gray.

Based on phylogenetic and sequence conservation analyses, GPCRs can be classified in 5 different families or classes (Fredriksson et al., 2003; Schioth and Fredriksson, 2005): rhodopsin (class A), secretin (class B1), adhesion (class B2), glutamate (class C), and frizzled/taste2 (class F). Nonetheless, taste 2 (or bitter taste) receptors have also been proposed to form part of class A (Nordstrom et al., 2011) or even constitute a sixth, additional family (class T) (Munk et al., 2016a). Since the appearance of the first crystal structure of rhodopsin in 2000, experimental structural characterization of GPCRs is blossoming (Munk et al., 2019). As of February 2019, there are 59 unique receptor structures solved (https://gpcrdb.org/structure/statistics), most of them corresponding to the rhodopsin (or class A) family (Figure 1). Molecular dynamics (MD) simulations started from these experimental structures have provided very important insights into ligand binding and receptor activation (Miao and McCammon, 2016; Sengupta et al., 2016; Latorraca et al., 2017; Marino and Filizola, 2018; Torrens-Fontanals et al., 2018; Velgy et al., 2018).

Nonetheless, the experimental structural coverage is still very far from the total of 800 GPCRs. In particular, there are no experimental structures available for three receptor groups: olfactory receptors (ORs, which constitute half of class A), taste 2 receptors (TAS2Rs, which represent the third largest GPCR family) and adhesion (class B2) receptors. *In silico* modeling can help to fill this gap of ~87% structurally uncharacterized GPCRs (Pándy-Szekeres et al., 2017). Indeed, the community-wide GPCR Dock assessment (Michino et al., 2009: Kufareva et al., 2011, 2014) has shown that homology modeling and ligand docking are able to provide valuable information on receptor-ligand interactions, in particular for those GPCR targets that have templates with sequence identity higher than 35–40% (Kufareva et al., 2011; Beuming and Sherman, 2012). Subsequent refinement of the bioinformatics-based models through molecular dynamics simulations (Kufareva et al., 2014; Cavasotto and Palomba, 2015; Lupala et al., 2018) and integration of experimental (mutagenesis and ligand structure-activity relationship) data (Munk et al., 2016b) further increases the model quality to values close to experimental accuracy. However, approximately *half* of GPCRs do not have a close template (i.e., an experimental structure of a receptor from the same family with a similar ligand). For instance, the sequence identity of 90% GPCRs with the rhodopsin template (representative of the largest GPCR family, class A) is lower than 20% (Zhang et al., 2006). Therefore, in most cases the *in silico* modeling approach needs further improvement, typically using molecular dynamics (Kufareva et al., 2014; Cavasotto and Palomba, 2015; Lupala et al., 2018).

Chemosensory receptors (olfactory and bitter taste receptors) are among the GPCRs without close templates. Increasing evidence shows that these receptors are expressed not only in the nose and the tongue, but also in other parts of the body (Foster et al., 2014; Abaffy, 2015; Ferrer et al., 2016; Shaik et al., 2016; Lu et al., 2017; Behrens and Meyerhof, 2019; Lee et al., 2019) and thus they have become attractive novel targets for drug design campaigns (Lee et al., 2019). However, chemosensory receptors represent a major challenge for computational modeling. Their sequence identity with the available GPCR templates is lower than 20% (Fierro et al., 2017) and thus only low resolution homology models can be generated (Kufareva et al., 2011; Fierro et al., 2017). Hence, our lab has made a major effort to attempt at improving such low resolution homology models and at making valuable predictions of the ligand binding determinants of these receptors. In this review, we first explain the computational approach used in our group to study low resolution GPCR models, based on the combination of state-of-the-art bioinformatics techniques and multiscale molecular dynamics simulations, as well as its validation on a class A GPCR (the β2-adrenergic receptor) with a solved crystallographic structure. Then, we show that, although bioinformatics-based models can be a good starting point to study receptor-ligand interactions, multiscale simulations significantly improve the quality of the models for which MM/CG simulations have been run so far. A perspective on this multiscale approach concludes this review.

## Materials and Methods

### Bioinformatics

Given the lack of experimental structures, the initial structures of the receptor/ligand complexes are generated using bioinformatics approaches. Although there are several webservers specialized in GPCR modeling (Launay et al., 2012; Zhang et al., 2015; Busato and Giorgetti, 2016; Esguerra et al., 2016; Pándy-Szekeres et al., 2017; Worth et al., 2017; Miszta et al., 2018), here we used the GOMoDo webserver (Sandal et al., 2013), which combines in a single pipeline homology modeling and molecular docking for GPCRs.

Since the sequence identity of any given olfactory or bitter taste receptor with the available GPCR templates is lower than 20% (Fierro et al., 2017), special care needs to be taken in the sequence alignment step. Hence, the alignment was done using profile Hidden Markov Models (HMMs) of the corresponding target receptor family and the GPCR template(s), which were generated with HHPred (Soding et al., 2005). This approach has been shown to improve the target-template alignment for distant homologs (Soding et al., 2005), in particular for GPCRs (Kufareva et al., 2014). This alignment was further improved by manual curation, taking advantage of the conserved seven transmembrane (7TM) helix topology and the presence of common conserved features across GPCRs (Lagerstrom and Schioth, 2008; Venkatakrishnan et al., 2013; Pydi et al., 2014, 2016; Tehan et al., 2014; de March et al., 2015; Di Pizio et al., 2016; Fierro et al., 2017). Moreover, since template selection is difficult with such low sequence identity, several models based on different templates were built using MODELLER (Webb and Sali, 2016), and the best model was selected considering also structural quality parameters (Melo et al., 2002; Shen and Sali, 2006).

The receptor structural model thus generated was then submitted to molecular docking using HADDOCK (Dominguez et al., 2003). Although other docking approaches were tested [based on AutoDock Vina (Trott and Olson, 2010) or Glide (Friesner et al., 2004)], no significant improvement in the quality of the models was observed (Fierro et al., 2017). The location of the ligand binding pocket inside the 7TM bundle is conserved (Venkatakrishnan et al., 2013), despite the low sequence identity among GPCRs. Moreover, the results of the GPCR Dock competitions (Michino et al., 2009; Kufareva et al., 2011, 2014; Cavasotto and Palomba, 2015; Munk et al., 2016b) seem to indicate that incorporating information about putative binding residues (from experimental data or computational predictions) helps to improve the docking results. Therefore, an information-driven approach was taken, in which the computationally predicted binding residues [using fpocket (Le Guilloux et al., 2009)] were used to guide the docking. Nonetheless, the fine details of the ligand binding site are expected to be highly variable across GPCRs (Venkatakrishnan et al., 2013), due to the chemical diversity of the GPCR ligands. Hence, in our HADDOCK-based docking approach both receptor and ligand were considered fully flexible in order to allow mutual readjustments. Other flexible docking approaches have also been successfully employed by other groups to predict the binding determinants of chemosensory receptors [see for instance (Di Pizio and Niv, 2014; Di Pizio et al., 2017; Xue et al., 2018)].

### Multiscale Molecular Dynamics Simulations

The results of the GPCR Dock competitions [reviewed in references (Cavasotto and Palomba, 2015) and (Ranganathan et al., 2017)] showed that refinement of the docked complexes using molecular dynamics simulations can significantly improve the prediction of receptor/ligand interactions. This is particularly important for GPCR models based on low sequence identity, as it is the case for chemosensory receptors, where the low accuracy of the side chain prediction and the limited sampling of the docking algorithms may undermine the quality of the bioinformatics-based models. There are several studies in the literature applying molecular dynamics simulations to chemosensory receptors (Gelis et al., 2012; Lai and Crasto, 2012; Charlier et al., 2013; Lai et al., 2014; Chen et al., 2018; Jaggupilli et al., 2018; Liu et al., 2018; Bushdid et al., 2019). Here we focus on a hybrid, multiscale approach developed in our group (Neri et al., 2005, 2008; Leguèbe et al., 2012; Giorgetti and Carloni, 2014; Musiani et al., 2015; Tarenzi et al., 2017), which is tailored to study ligand binding in GPCRs.

**Figure 2:**
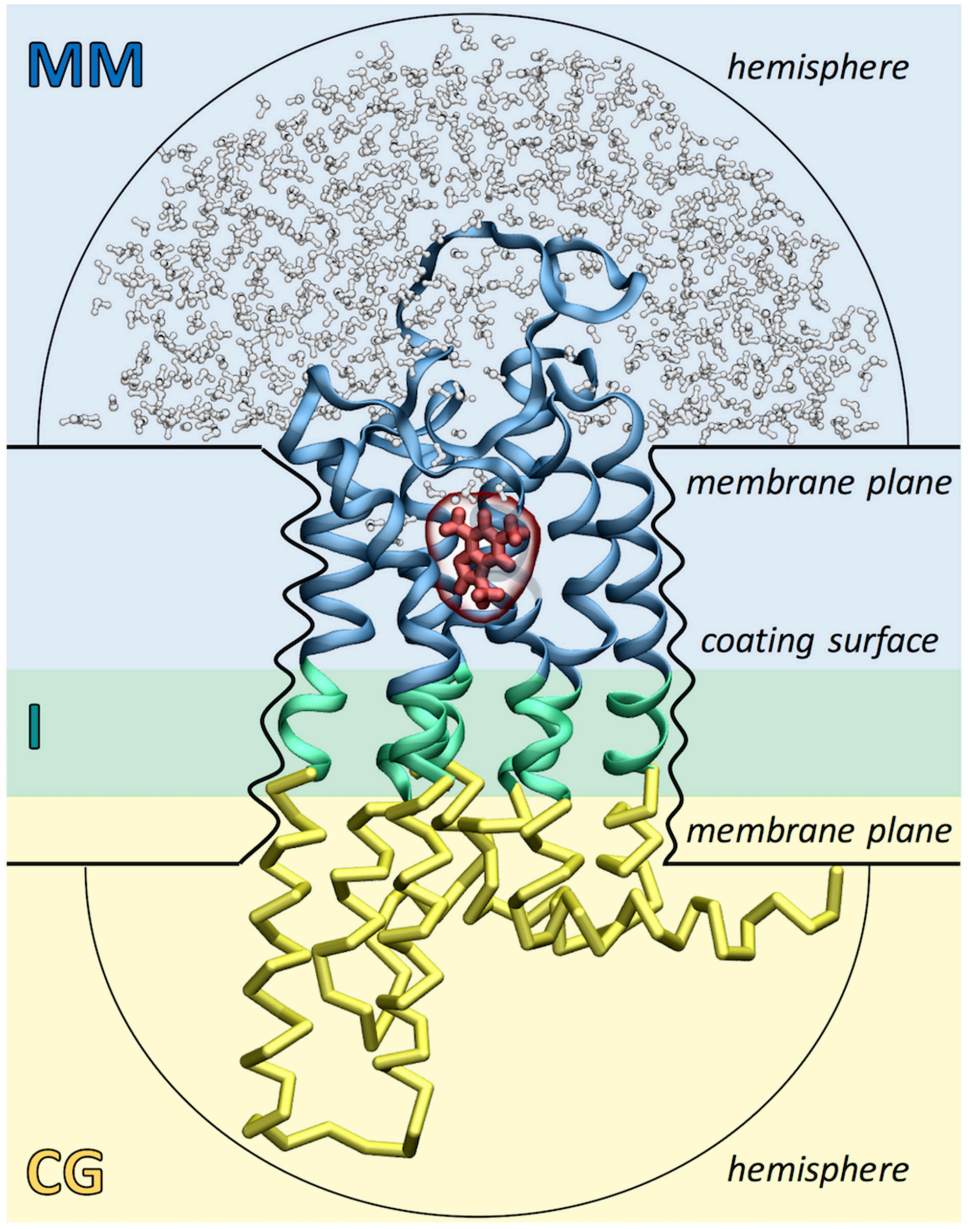
Simulation setup of the Molecular Mechanics/Coarse-Grained (MM/CG) approach. The receptor (GPCR) is divided in three regions: the extracellular MM part (in blue), the intracellular CG part (in yellow) and the connecting interface (I, in green). The ligand (shown in red) and the surrounding receptor residues and water molecules (in blue and white, respectively) are described with MM. The presence of the lipid bilayer is modeled implicitly by incorporating three different wall potentials (upper and lower membrane planes and coating surface) and two additional hemispheric walls are included to cap the ends of the protein and prevent water evaporation.

As shown in Figure 2, the ligand, the surrounding protein residues (typically the extracellular half of the receptor) and water molecules are described with molecular mechanics (MM) using the GROMOS united-atom force field (Schuler and Van Gunsteren, 2000; Schuler et al., 2001; Oostenbrink et al., 2004). Instead, the rest of the protein (i.e., the intracellular half of the receptor) is treated at the coarse grained (CG) level using a Gō model (Go and Abe, 1981). Each amino acid is mapped into a single coarse grained bead corresponding to the alpha carbon atom and native contacts are mimicked by introducing two new potential terms. The bonded interactions between consecutive CG beads are taken into account using a quartic potential, whereas the non-bonded interactions between non-consecutive CG beads are described through a Morse-like potential. The MM and CG regions are connected by an interface (I) region, which ensures the continuity of the protein backbone by coupling the two levels of resolution. The MM-I interaction is treated at the atomistic level using the GROMOS force field, whereas the I-CG interaction is described using the Gō model. Namely, bonded interactions are calculated between the Cα atoms of the I residues and the consecutive CG beads, whereas non-bonded interactions are computed using both the Cα and Cβ atoms of the I residues and the non-consecutive CG beads.

The presence of the lipid bilayer is modeled implicitly, using three wall potentials: a “coating surface” wall that simulates the effect of the lipid hydrophobic tails embracing the protein surface and two “membrane plane” walls that mimic the presence of the lipid head groups. In addition, two “hemispheric” wall potentials are included to cap the extracellular and cytoplasmic ends of the protein and to prevent water evaporation. Water molecules, Cα atoms and aromatic residues Phe, Trp, and Tyr (the so-called “anchor residues”) are affected by these boundary potentials, which are added to the MM/CG potential energy function as functions of the distance of an atom to the closest wall. Recently, a reservoir of CG water has been introduced around the MM water cap, permitting water molecules to freely diffuse between the MM and CG regions, changing *on the fly* their resolution. This allows to carry out simulations in a statistically well-defined (grand canonical) ensemble in the higher-resolution MM region, resulting in a further improved description of the binding poses and the binding site flexibility (Tarenzi et al., 2019).

Compared to docking, these multiscale simulations allow to (i) sample protein flexibility and protein/ligand interactions more extensively (~1 μs timescale) and (ii) include explicit water molecules, which may be involved in ligand binding in GPCRs (Pardo et al., 2007; Angel et al., 2009; Venkatakrishnan et al., 2019). Moreover, the use of the Go model in the intracellular half of the receptor prevents possible unfolding problems due the initial wrong orientation of the side chains in the low resolution homology model. For further details on the MM/CG implementation, we refer the reader to some recent reviews (Giorgetti and Carloni, 2014; Musiani et al., 2015; Schneider et al., 2018).

## Results and Discussion

### Validation of the Molecular Mechanics/Coarse Grained (MM/CG) Approach

The reliability of the MM/CG approach was assessed using the β2-adrenergic receptor (β2-AR) in complex with either its inverse agonist *S*-carazolol or its agonist *R*-isoprenaline (Leguèbe et al., 2012; Marchiori et al., 2013). The availability of a crystal structure of the receptor (for the first complex) (Cherezov et al., 2007), as well all-atom (AA) molecular dynamics simulations (for both complexes) (Vanni et al., 2011) allows to compare the results of the MM/CG simulations with both static and dynamical data. Three different types of tests were carried out (Leguèbe et al., 2012; Marchiori et al., 2013), started from different initial structures: (i) the same initial structures of the β2-AR/*S*-carazolol and β2-AR/*R*-isoprenaline complexes as the atomistic simulations, (ii) an alternative initial structure of the β2-AR/*S*-carazolol complex built by displacing the ligand to a position where none of the crystallographic receptor/ligand interactions was present, and (iii) a low resolution model of the β2-AR/*S*-carazolol complex built using bioinformatics. Each of the test simulations were ~0.8 μs long.

The first test (Leguèbe et al., 2012) showed that the MM/CG approach is able to preserve the receptor/ligand complex structure observed in the crystal structure, as well as to provide dynamical and hydration information similar to the AA simulations, but at a lower computational cost. Complementarily, the second test (Leguèbe et al., 2012) confirmed that the agreement between the MM/CG and AA simulations observed in the first test is not due to the use of a common initial structure and, furthermore, demonstrated the predictive power of the MM/CG approach even when starting from a wrong binding pose. Nonetheless, the two previous tests can be considered as redocking experiments: even though the system was converted from AA into hybrid MM/CG [test (i)] or the ligand was moved out of place [test (ii)], the binding residues were already positioned as in the correct binding pose. Instead, the third test (Marchiori et al., 2013) validated the reliability of the MM/CG approach applied to low resolution models, as the ones used for the bitter taste receptors discussed in the next section. In such models, the orientation of the side chains is expected to be hardly accurate, due to the low sequence identity with the template used in the homology modeling (Chothia and Lesk, 1986; Baker and Sali, 2001; Eramian et al., 2008; Olivella et al., 2013; Piccoli et al., 2013; Busato and Giorgetti, 2016). Indeed, the homology model of the β2-adrenergic receptor (built using as template the experimental structure of squid rhodopsin) shares only a 20% sequence identity with the target and thus docking of the ligand *S*-carazolol resulted in a wrong binding pose. However, the ~0.8 μs MM/CG simulation is able to yield a binding pose showing receptor/ligand interactions similar to the crystallographic ones (Marchiori et al., 2013).

### Predictive Power of the Computational Models of Chemosensory Receptors

In order to investigate the performance of bioinformatics and multiscale simulations in predicting receptor/ligand interactions in chemosensory receptors, we selected those receptor/ligand pairs for which experimental data are available (Fierro et al., 2017). As of August 2017, these included seven olfactory receptor/odorant complexes and fifteen bitter taste receptor/bitter tastant complexes with available site-directed mutagenesis data and functional assays, typically agonist dose-response curves. The docked receptor/ligand complexes were obtained using the bioinformatics protocol described in the Materials and Methods section, whereas the three MM/CG simulations analyzed (for the complexes TAS2R38/6-n-propylthiouracil, TAS2R38/phenylthiocarbamide and TAS2R46/strychnine) were taken from previous studies from our group (Biarnés et al., 2010; Marchiori et al., 2013; Sandal et al., 2015).

In order to compare the computational models with the experimental data, we defined “computational binding” and “computational non-binding” residues, as well as “experimental binding” and “experimental non-binding” residues (Fierro et al., 2017). Computational binding and non-binding residues were determined based on the receptor/ligand distance (using a cutoff of 5.5 Å) and the presence or absence (respectively) of an actual chemical interaction (such as hydrogen bonds, salt bridges, hydrophobic or aromatic interactions, etc.). Experimental binding residues were inferred from experiments based on (i) the effect of the mutation on the half maximal effective concentration (EC_50_) value and (ii) their position in the upper extracellular part of the receptor, where the canonical binding site of class A GPCRs is located (Venkatakrishnan et al., 2013). Residues whose mutation does not change EC_50_ and/or that are located in the lower intracellular part of the receptor are considered as experimental non-binding residues. Obviously, this is a simplistic definition of binding residue, as from the experimental data we cannot discard that these residues might also be involved in receptor activation (see reference Fierro et al., 2017 for further discussion).

Comparison of the computational and experimental residues yielded four different possible test outcomes. “True positives” (TP) were amino acids identified as binding residues by both experiment and computation, “false positives” (FP) were amino acids identified as non-binding residues by experiment but as binding residues in computation, “true negatives” (TN) were amino acids identified as non-binding residues by both experiment and computation, and “false negatives” (FN) were amino acids identified as binding residues by experiment but not in computation. In order to assess the agreement of the computational models with the experimental data, two statistical parameters, precision (PREC) and recall (REC), were calculated:

$$\begin{array}{lll} \text{PREC} & = & \text{TP}/\left(\text{TP}+\text{FP}\right)\\ \text{ REC} & = & \text{TP}/\left(\text{TP}+\text{FN}\right) \end{array}$$

These parameters are close to 1 when the computational predictions were consistent with the experimental data, and zero when they were not. Precision and recall values were calculated for the best docking poses of the twenty-two complexes investigated and for a representative snapshot of each of the three MM/CG simulations analyzed (Fierro et al., 2017).

We found that the predictive power of the bioinformatics approach varied from complex to complex. Nonetheless, the general agreement between the binding residues identified in the docking poses and those inferred from experiments was low, with only 36% of the predictions consistent with experiment (Fierro et al., 2017). Residues shown experimentally to be important for binding were not observed in the docked complexes (i.e., low recall) and/or residues not involved in protein/ligand interactions according to experiments were predicted as binding residues by computation (i.e., low precision). Most likely, this is due, among other factors, to the low sequence identity (<20%) between the chemosensory receptor targets and the available GPCR templates, as well as the limited sampling of the docking algorithms. Therefore, although the bioinformatics-based models are a good starting point to study ligand binding determinants in chemosensory receptors, they appear to require further refinement (Fierro et al., 2017). This finding is consistent with the results of the GPCR Dock competitions, which indicated that models based on sequence identity below 30% need substantial improvement in order to reach accuracy comparable to experimental structures (Kufareva et al., 2011, 2014).

Next, we compared the performance of molecular dynamics for the three bitter taste receptor complexes studied so far with (~0.8–1 μs long) MM/CG simulations (Marchiori et al., 2013; Sandal et al., 2015). We found that the predictive power of the computational models improved dramatically, with 96–100% of the predictions in agreement with experiment (Fierro et al., 2017). Most residues shown to be involved in binding by experiments are captured by the MM/CG simulations and the number of wrong predictions was minimal (i.e., both recall and precision increased to values near or equal to one, see Table 1). Considering the nearly 20 mutants tested experimentally for either TAS2R38 (Biarnés et al., 2010: Marchiori et al., 2013) or TAS2R46 (Brockhoff et al., 2010; Born et al., 2013; Sandal et al., 2015), the agreement of the computational models with experiments seems really remarkable. Moreover, although in our analysis we used all the available mutagenesis data to validate *a posteriori* the MM/CG results, simulations were also able to predict new binding residues. Indeed, the simulations of the TAS2R38 and TAS2R46 complexes suggested several binding residues not tested previously and these predictions were subsequently verified by performing additional mutagenesis and functional assays (Marchiori et al., 2013; Sandal et al., 2015). Altogether, multiscale simulations seem to be a robust approach for identifying ligand binding residues in olfactory and bitter taste receptors, at least for the three bitter taste receptor complexes studied so far (Fierro et al., 2017). This is consistent with the conclusions of the GPCR Dock competitions, where molecular dynamics simulations and integration of experimental data (such as site-directed mutagenesis or ligand structure-activity relationships) were shown to improve the computational predictions (Kufareva et al., 2011, 2014; Cavasotto and Palomba, 2015; Ranganathan et al., 2017). Nonetheless, the application of MM/CG simulations to other chemosensory receptor complexes with available experimental data is still needed to firmly establish the reliability and transferability of this method.

**Table 1:** Evaluation of the performance of the bioinformatics models and the multiscale MM/CG simulations for the three bitter taste receptors studied so far, i.e. TAS2R38/6-n-propylthiouracil (PROP), TAS2R38/phenylthiocarbamide (PTC) and TAS2R46/strychnine (Fierro et al., 2017).

	**TAS2R38/PROP**		**TAS2R38/PTC**		**TAS2R46/strychnine**	
	**Model**	**MM/CG**	**Model**	**MM/CG**	**Model**	**MM/CG**
PREC	0	1	0	0.75	0.60	1
REC	0	1	0	1	0.33	1

*Precision (PREC) and recall (REC) values are listed*.

### Insights Into Ligand Selectivity Determinants in Bitter Taste Receptors

There are around 1,000 compounds characterized as bitter, which vary significantly in size, polarity and chemical structure (Meyerhof et al., 2010; Behrens and Meyerhof, 2018; Dagan-Wiener et al., 2019). To make things even more puzzling, three receptors (TAS2R10, TAS2R14, and TAS2R46) out of the 25 bitter taste receptors are able to recognize about half of these bitter compounds (Behrens and Meyerhof, 2018). In contrast to this broad agonist spectrum, there are two receptors, TAS2R38 and TAS2R16, that are specialized in detecting a specific chemical group (thiourea/isothiocyanate and β-D-glucopyranoside, respectively) (Behrens and Meyerhof, 2018). Here we discuss in detail the structural predictions described above to investigate whether they can help understand the molecular basis of this disparate ligand selectivity. In particular, the MM/CG approach has been applied so far to one receptor of each group, i.e., TAS2R46 (Sandal et al., 2015) and TAS2R38 (Biarnés et al., 2010; Marchiori et al., 2013).

**Figure 3:**
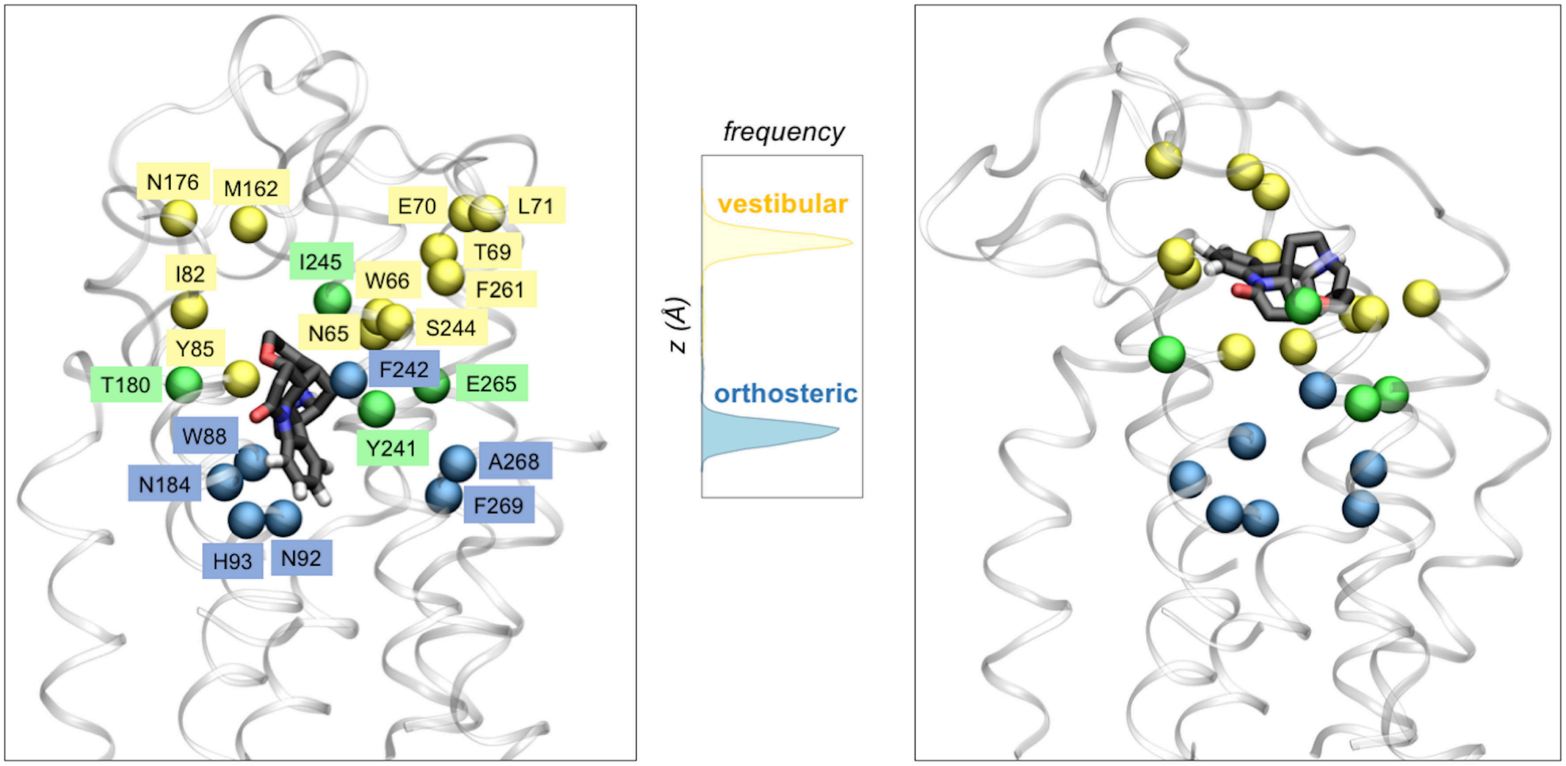
Two binding site architecture of TAS2R46. The agonist strychnine (in licorice representation) can bind in either the orthosteric site (left panel) or the vestibular site (right panel). Receptor residues interacting with the ligand in the orthosteric site, the vestibular site or both sites are shown with blue, yellow or green spheres, respectively. The central panel displays the distribution of the ligand center-of-mass z coordinate for the two ~1 μs MM/CG simulations, showing that strychnine stabilized either in the orthosteric site or in a second (vestibular) site, located further toward the extracellular side.

The microsecond-long simulations of TAS2R46 in complex with its agonist strychnine (Sandal et al., 2015) showed that the ligand can explore not only one but two different binding cavities (Figure 3). The first one coincides with the canonical binding site of class A GPCRs (i.e., the so-called orthosteric site), whereas the second is located further toward the extracellular side and thus has been denoted as “vestibular.” The mutagenesis data is compatible with this two-site architecture, as the residues experimentally inferred to be involved in binding (Brockhoff et al., 2010; Born et al., 2013; Sandal et al., 2015) are distributed between the two sites (Figure 3). Moreover, the identified vestibular binding cavity overlaps with the extracellular allosteric binding site observed for class A GPCRs (Dror et al., 2011, 2013; Kruse et al., 2012; Abdul-Ridha et al., 2014; Latorraca et al., 2017; Thal et al., 2018), further supporting its existence. This two-step binding architecture may constitute the molecular basis of the “access control” mechanism proposed by Meyerhof and coworkers (Brockhoff et al., 2010) and would help TAS2R46 to discriminate the wide range of ligands recognized by this promiscuous receptor (Sandal et al., 2015). Moreover, a bioinformatics analysis of the binding residues predicted for TAS2R46 across the bitter taste receptor family showed that half of these functionally relevant positions are conserved in two or more TAS2Rs, suggesting that the vestibular site might also be present in other receptors of this family (Sandal et al., 2015). However, the ~0.8 μs simulations of TAS2R38 in complex with either PTC or PROP showed the ligand bound in a single site, corresponding to the orthosteric one (Marchiori et al., 2013). This hints at the possibility that the vestibular site is not as crucial for a group specific receptor such as TAS2R38 or even that the two-site architecture is not required for a more selective receptor (Suku et al., 2017). Naturally, given the crudeness of our models, further simulations and experimental studies on other members of the bitter taste receptor family are needed in order to confirm this proposal.

## Conclusions

**Figure 4:**
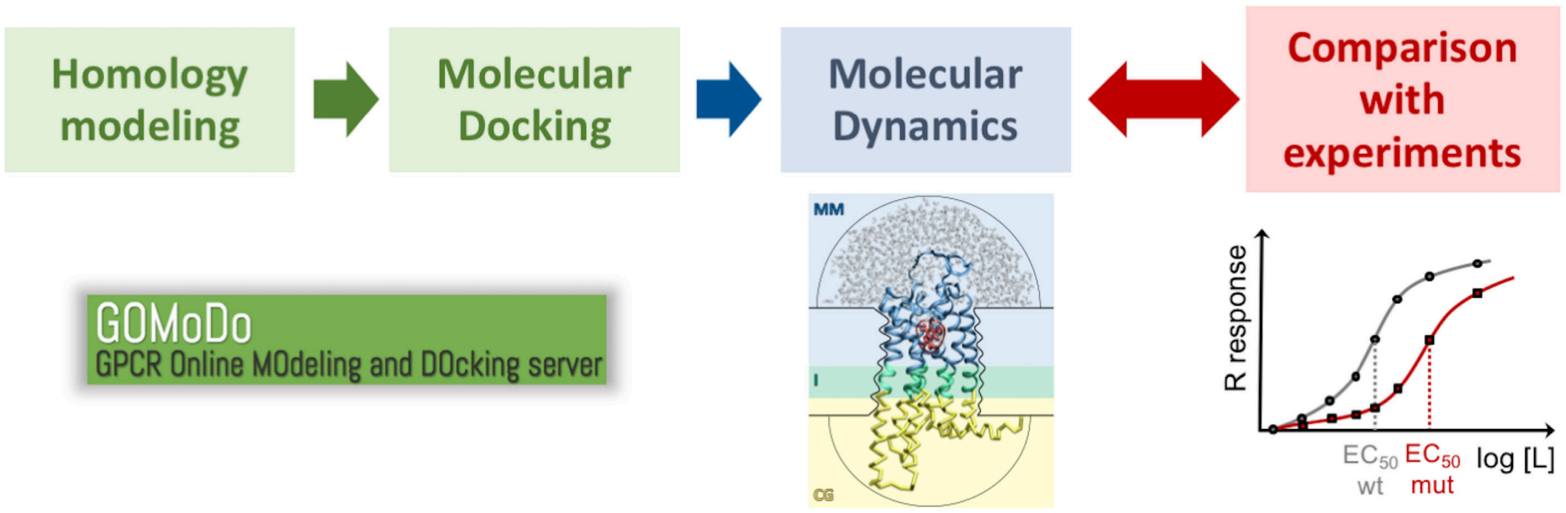
Proposed protocol to study ligand binding in low resolution GPCR models. The initial model generated with homology modeling and molecular docking (e.g., using the GOMoDo webserver, Sandal et al., 2013) is further refined using molecular dynamics (e.g., using the molecular mechanics/coarse grained or MM/CG approach developed in our group, Leguèbe et al., 2012). The receptor/ligand interactions predicted by the simulations have to be validated by extensive comparison with experimental data (typically site-directed mutagenesis and dose-response functional assays).

Given the scarcity of experimental structural data (Munk et al., 2019), computational modeling of GPCRs is essential to understand ligand binding and design new drugs targeting this biologically and pharmacologically relevant family (Michino et al., 2009; Kufareva et al., 2011, 2014; Cavasotto and Palomba, 2015; Ranganathan et al., 2017; Lupala et al., 2018). These computational approaches (Figure 4) include homology modeling and molecular docking, often supplemented with experimental (mutagenesis and ligand structure-activity relationship) data. Subsequent refinement with molecular dynamics simulations has been shown to further improve the computational predictions (Kufareva et al., 2014; Cavasotto and Palomba, 2015; Ranganathan et al., 2017; Lupala et al., 2018). The accuracy of the models thus generated might reach values near the experimental ones for those GPCRs with a close structural template (i.e., with sequence identity larger than 35–40% and a chemically similar ligand) (Kufareva et al., 2011; Beuming and Sherman, 2012). However, for most GPCRs the closest structural template has sequence identity below this threshold, and thus computational predictions become challenging. This the case for olfactory and bitter taste receptors, which constitute the first and third largest GPCR groups, respectively, as their sequence identity with the available GPCR templates is below 20%.

In this review, we have shown that molecular dynamics simulations, in particular the multiscale molecular mechanics / coarse grained approach developed in our group (Neri et al., 2005, 2008; Leguèbe et al., 2012; Giorgetti and Carloni, 2014; Musiani et al., 2015; Tarenzi et al., 2017), can overcome, at least in part, these limitations (Fierro et al., 2017) and successfully predict residues involved in ligand binding for the three bitter taste receptor complexes studied so far (Biarnés et al., 2010; Marchiori et al., 2013; Sandal et al., 2015). The natural extension of these previous works would be to other bitter taste and olfactory receptors for which experimental data are available. In addition, MM/CG simulations could be easily applied to other GPCRs. Although this approach has been used so far for a limited number of GPCR/ligand complexes (Leguèbe et al., 2012; Marchiori et al., 2013; Sandal et al., 2015), the excellent agreement of the computationally predicted binding poses with the experimental mutagenesis data [for the aforementioned three bitter taste receptor complexes (Marchiori et al., 2013; Sandal et al., 2015)] or the crystal structure [for the β2-adrenergic receptor (Leguèbe et al., 2012)] further supports the applicability of the MM/CG method to other GPCR/ligand complexes. Indeed, MM/CG simulations have been recently used to model the synthetic agonist diphenyleneiodonium chloride (DPI) bound to its target receptor GPR3 (Capaldi et al., 2018). Two of the predicted DPI binding residues were successfully validated *a posteriori* using mutagenesis and functional assays, as previously done for TAS2R38 (Marchiori et al., 2013) and TAS2R46 (Sandal et al., 2015).

## Data Availability

The raw data supporting the conclusions of this manuscript will be made available by the authors, without undue reservation, to any qualified researcher.

## Author Contributions

All authors listed have made a substantial, direct and intellectual contribution to the work, and approved it for publication.

### Conflict of Interest Statement

LN was employed by the company illycafè S.p.A (Trieste, Italy). The remaining authors declare that the research was conducted in the absence of any commercial or financial relationships that could be construed as a potential conflict of interest.

## References

[B1] Abaffy T. (2015). Human olfactory receptors expression and their role in non- olfactory tissues-a mini-review. J. Pharmacogenom. Pharmacoproteom. 6:1 10.4172/2153-0645.1000152

[B2] Abdul-Ridha A. López L. Keov P. Thal D. M. Mistry S. N. Sexton P. M.. (2014). Molecular determinants of allosteric modulation at the M1 muscarinic acetylcholine receptor. J. Biol. Chem. 289, 6067–6079. 10.1074/jbc.M113.539080 24443568 PMC3937673

[B3] Alexander S. P. H. Kelly E. Marrion N. V. Peters J. A. Faccenda E. Harding S. D. Collaborators. (2017). The Concise Guide to PHARMACOLOGY 2017/18: G protein-coupled receptors. Br. J. Pharmacol. 174, S17–S129. 10.1111/bph.13878 29055040 PMC5650667

[B4] Angel T. E. Chance M. R. Palczewski K. (2009). Conserved waters mediate structural and functional activation of family A (rhodopsin-like) G protein-coupled receptors. Proc. Natl. Acad. Sci. U.S.A. 106, 8555–8560. 10.1073/pnas.0903545106 19433801 PMC2688986

[B5] Baker D. Sali A. (2001). Protein structure prediction and structural genomics. Science 294, 93–96. 10.1126/science.1065659 11588250

[B6] Behrens M. Meyerhof W. (2018). Vertebrate bitter taste receptors: keys for survival in changing environments. J. Agric. Food Chem. 66, 2204–2213. 10.1021/acs.jafc.6b04835 28013542

[B7] Behrens M. Meyerhof W. (2019). A role for taste receptors in (neuro)endocrinology? J. Neuroendocrinol. 31:e12691. 10.1111/jne.12691 30712315

[B8] Beuming T. Sherman W. (2012). Current assessment of docking into GPCR crystal structures and homology models: successes, challenges, and guidelines. J. Chem. Inf. Model. 52, 3263–3277. 10.1021/ci300411b 23121495

[B9] Biarnés X. Marchiori A. Giorgetti A. Lanzara C. Gasparini P. Carloni P.. (2010). Insights into the binding of Phenyltiocarbamide (PTC) agonist to its target human TAS2R38 bitter receptor. PLoS ONE 5:e12394. 10.1371/journal.pone.0012394 20811630 PMC2928277

[B10] Born S. Levit A. Niv M. Y. Meyerhof W. Behrens M. (2013). The human bitter taste receptor TAS2R10 is tailored to accommodate numerous diverse ligands. J. Neurosci. 33, 201–213. 10.1523/JNEUROSCI.3248-12.2013 23283334 PMC6618634

[B11] Brockhoff A. Behrens M. Niv M. Y. Meyerhof W. (2010). Structural requirements of bitter taste receptor activation. Proc. Natl. Acad. Sci. U.S.A. 107, 11110–11115. 10.1073/pnas.0913862107 20534469 PMC2890741

[B12] Busato M. Giorgetti A. (2016). Structural modeling of G-protein coupled receptors: an overview on automatic web-servers. Int. J. Biochem. Cell Biol. 77, 264–274. 10.1016/j.biocel.2016.04.004 27102413

[B13] Bushdid C. Claire A. Topin J. Do M. Matsunami H. Golebiowski J. (2019). Mammalian class I odorant receptors exhibit a conserved vestibular-binding pocket. Cell. Mol. Life Sci. 76, 995–1004. 10.1007/s00018-018-2996-4 30599066 PMC7313674

[B14] Capaldi S. Suku E. Antolini M. Di Giacobbe M. Giorgetti A. Buffelli M. (2018). Allosteric sodium binding cavity in GPR3: a novel player in modulation of Aβ production. Sci. Rep. 8:11102. 10.1038/s41598-018-29475-7 30038319 PMC6056553

[B15] Cavasotto C. N. Palomba D. (2015). Expanding the horizons of G protein-coupled receptor structure-based ligand discovery and optimization using homology models. Chem. Commun. 51, 13576–13594. 10.1039/C5CC05050B 26256645

[B16] Charlier L. Topin J. de March C. A. Lai P. C. Crasto C. J. Golebiowski J. (2013). Molecular modelling of odorant/olfactory receptor complexes. Methods Mol. Biol. 1003, 53–65. 10.1007/978-1-62703-377-0_4 23585033

[B17] Chen Z. Dong S. Meng F. Liang Y. Zhang S. Sun J. (2018). Insights into the binding of agonist and antagonist to TAS2R16 receptor: a molecular simulation study. Mol. Simul. 44, 322–329. 10.1080/08927022.2017.1376325

[B18] Cherezov V. Rosenbaum D. M. Hanson M. A. Rasmussen S. G. F. Thian F. S.. (2007). High-resolution crystal structure of an engineered human beta2-adrenergic G protein coupled receptor. Science 318, 1258–1265. 10.1126/science.1150577 17962520 PMC2583103

[B19] Chothia C. Lesk A. M. (1986). The relation between the divergence of sequence and structure in proteins. EMBO J. 5, 823–826. 3709526 10.1002/j.1460-2075.1986.tb04288.x PMC1166865

[B20] Dagan-Wiener A. Di Pizio A. Nissim I. Bahia M. S. Dubovski N. Margulis E.. (2019). BitterDB: taste ligands and receptors database in 2019. Nucleic Acids Res. 47, D1179–D1185. 10.1093/nar/gky974 30357384 PMC6323989

[B21] de March C. A. Kim S. K. Antonczak S. Goddard W. A. I. I. I. Golebiowski J. (2015). G protein-coupled odorant receptors: from sequence to structure. Protein Sci. 24, 1543–1548. 10.1002/pro.2717 26044705 PMC4570547

[B22] Di Pizio A. Kruetzfeldt L. M. Cheled-Shoval S. Meyerhof W. Behrens M. Niv M. Y. (2017). Ligand binding modes from low resolution GPCR models and mutagenesis: chicken bitter taste receptor as a test-case. Sci. Rep. 7:8223. 10.1038/s41598-017-08344-9 28811548 PMC5557796

[B23] Di Pizio A. Levit A. Slutzki M. Behrens M. Karaman R. Niv M. Y. (2016). Comparing class A GPCRs to bitter taste receptors: structural motifs, ligand interactions and agonist-to-antagonist ratios. Methods Cell Biol. 132, 401–427. 10.1016/bs.mcb.2015.10.005 26928553

[B24] Di Pizio A. Niv M. Y. (2014). Computational studies of smell and taste receptors. Isr. J. Chem. 54, 1205–1218. 10.1002/ijch.201400027

[B25] Dominguez C. Boelens R. Bonvin A. M. (2003). HADDOCK: a protein-protein docking approach based on biochemical or biophysical information. J. Am. Chem. Soc. 125, 1731–1737. 10.1021/ja026939x 12580598

[B26] Dror R. O. Green H. F. Valant C. Borhani D. W. Valcourt J. R. Pan A. C.. (2013). Structural basis for modulation of a G-protein-coupled receptor by allosteric drugs. Nature 503, 295–299. 10.1038/nature12595 24121438

[B27] Dror R. O. Pan A. C. Arlow D. H. Borhani D. W. Maragakis P. Shan Y.. (2011). Pathway and mechanism of drug binding to G-protein-coupled receptors. Proc. Natl. Acad. Sci. U.S.A. 108, 13118–13123. 10.1073/pnas.1104614108 21778406 PMC3156183

[B28] Eramian D. Eswar N. Shen M. Y. Sali A. (2008). How well can the accuracy of comparative protein structure models be predicted? Protein Sci. 17, 1881–1893. 10.1110/ps.036061.108 18832340 PMC2578807

[B29] Esguerra M. Siretskiy A. Bello X. Sallander J. Gutiérrez-de-Terán H. (2016). GPCR-ModSim: a comprehensive web based solution for modeling G-protein coupled receptors. Nucleic Acids Res. 44, W455–W462. 10.1093/nar/gkw403 27166369 PMC4987938

[B30] Ferrer I. Garcia-Esparcia P. Carmona M. Carro E. Aronica E. Kovacs G. G.. (2016). Olfactory receptors in non-chemosensory organs: the nervous system in health and disease. Front. Aging Neurosci. 8:163. 10.3389/fnagi.2016.00163 27458372 PMC4932117

[B31] Fierro F. Suku E. Alfonso-Prieto M. Giorgetti A. Cichon S. Carloni P. (2017). Agonist binding to chemosensory receptors: a systematic bioinformatics analysis. Front. Mol. Biosci. 4:63. 10.3389/fmolb.2017.00063 28932739 PMC5592726

[B32] Foster S. R. Roura E. Thomas W. G. (2014). Extrasensory perception: odorant and taste receptors beyond the nose and mouth. Pharmacol. Ther. 142, 41–61. 10.1016/j.pharmthera.2013.11.004 24280065

[B33] Fredriksson R. Lagerstrom M. C. Lundin L. G. Schioth H. B. (2003). The G-protein-coupled receptors in the human genome form five main families. Phylogenetic analysis, paralogon groups, and fingerprints. Mol. Pharmacol. 63, 1256–1272. 10.1124/mol.63.6.1256 12761335

[B34] Friesner R. A. Banks J. L. Murphy R. B. Halgren T. A. Klicic J. J. Mainz D. T.. (2004). Glide: a new approach for rapid, accurate docking and scoring. 1. Method and assessment of docking accuracy. J. Med. Chem. 47, 1739–1749. 10.1021/jm0306430 15027865

[B35] Gelis L. Wolf S. Hatt H. Neuhaus E. M. Gerwert K. (2012). Prediction of a ligand-binding niche within a human olfactory receptor by combining site-directed mutagenesis with dynamic homology modeling. Angew. Chem. Int. Ed Engl. 51, 1274–1278. 10.1002/anie.201103980 22144177

[B36] Giorgetti A. Carloni P. (2014). Molecular mechanics/coarse-grained models, in Protein Modelling, ed Naray-Szabo G. (Cham: Springer), 165–174. 10.1007/978-3-319-09976-7_7

[B37] Go N. Abe H. (1981). Noninteracting local-structure model of folding and unfolding transition in globular proteins. I. Formulation. Biopolymers 20, 991–1011. 10.1002/bip.1981.360200511 7225531

[B38] Hauser A. S. Attwood M. M. Rask-Andersen M. Schiöth H. B. Gloriam D. E. (2017). Trends in GPCR drug discovery: new agents, targets and indications. Nat. Rev. Drug Disc. 16, 829–842. 10.1038/nrd.2017.178 29075003 PMC6882681

[B39] Hauser A. S. Chavali S. Masuho I. Jahn L. J. Martemyanov K. A. Gloriam D. E.. (2018). Pharmacogenomics of GPCR drug targets. Cell 172, 41–54. 10.1016/j.cell.2017.11.033 29249361 PMC5766829

[B40] Jaggupilli A. Singh N. De Jesus V. C. Gounni M. S. Dhanaraj P. Chelikani P. (2018). Chemosensory bitter taste receptors (T2Rs) are activated by multiple antibiotics. FASEB J. 33, 501–517. 10.1096/fj.201800521RR 30011231

[B41] Kruse A. C. Hu J. Pan A. C. Arlow D. H. Rosenbaum D. M. Rosemond E.. (2012). Structure and dynamics of the M3 muscarinic acetylcholine receptor. Nature 482, 552–556. 10.1038/nature10867 22358844 PMC3529910

[B42] Kufareva I. Katritch V. Participants of G. D. Stevens R. C. Abagyan R. (2014). Advances in GPCR modeling evaluated by the GPCR Dock 2013 assessment: meeting new challenges. Structure 22, 1120–1139. 10.1016/j.str.2014.06.012 25066135 PMC4126895

[B43] Kufareva I. Rueda M. Katritch V. Stevens R. C. Abagyan R. Participants G. D. (2011). Status of GPCR modeling and docking as reflected by community-wide GPCR Dock 2010 assessment. Structure 19, 1108–1126. 10.1016/j.str.2011.05.012 21827947 PMC3154726

[B44] Lagerstrom M. C. Schioth H. B. (2008). Structural diversity of G protein- coupled receptors and significance for drug discovery. Nat. Rev. Drug Discov. 7, 339–357. 10.1038/nrd2518 18382464

[B45] Lai P. C. Crasto C. J. (2012). Beyond modeling: all-atom olfactory receptor model simulations. Front. Genet. 3:61. 10.3389/fgene.2012.00061 22563330 PMC3342527

[B46] Lai P. C. Guida B. Shi J. Crasto C. J. (2014). Preferential binding of an odor within olfactory receptors: a precursor to receptor activation. Chem. Senses 39, 107–123. 10.1093/chemse/bjt060 24398973 PMC3894857

[B47] Latorraca N. R. Venkatakrishnan A. J. Dror R. O. (2017). GPCR dynamics: structures in motion. Chem. Rev. 117, 139–155. 10.1021/acs.chemrev.6b00177 27622975

[B48] Launay G. Téletchéa S. Wade F. Pajot-Augy E. Gibrat J. F. Sanz G. (2012). Automatic modeling of mammalian olfactory receptors and docking of odorants. Protein Eng. Des. Sel. 25, 377–386. 10.1093/protein/gzs037 22691703

[B49] Le Guilloux V. Schmidtke P. Tuffery P. (2009). Fpocket: an open source platform for ligand pocket detection. BMC Bioinform. 10:168. 10.1186/1471-2105-10-168 19486540 PMC2700099

[B50] Lee S. J. Depoortere I. Hatt H. (2019). Therapeutic potential of ectopic olfactory and taste receptors. Nat. Rev. Drug Discov. 18, 116–138. 10.1038/s41573-018-0002-3 30504792

[B51] Leguèbe M. Nguyen C. Capece L. Hoang Z. Giorgetti A. Carloni P. (2012). Hybrid molecular mechanics/coarse-grained simulations for structural prediction of G-protein coupled receptor/ligand complexes. PLoS ONE 7:e47332. 10.1371/journal.pone.0047332 23094046 PMC3477165

[B52] Liu K. Jaggupilli A. Premnath D. Chelikani P. (2018). Plasticity of the ligand binding pocket in the bitter taste receptor T2R7. Biochim. Biophys. Acta Biomembr. 1860, 991–999. 10.1016/j.bbamem.2018.01.014 29355483

[B53] Lu P. Zhang C. H. Lifshitz L. M. ZhuGe R. (2017). Extraoral bitter taste receptors in health and disease. J. Gen. Physiol. 149, 181–197. 10.1085/jgp.201611637 28053191 PMC5299619

[B54] Lupala C. S. Rasaeifar B. Gomez-Gutierrez P. Perez J. J. (2018). Using molecular dynamics for the refinement of atomistic models of GPCRs by homology modeling. J. Biomol. Struct. Dyn. 36, 2436–2448. 10.1080/07391102.2017.1357503 28728517

[B55] Marchiori A. Capece L. Giorgetti A. Gasparini P. Behrens M. Carloni P.. (2013). Coarse-grained/molecular mechanics of the TAS2R38 bitter taste receptor: experimentally-validated detailed structural prediction of agonist binding. PLoS ONE 8:e64675. 10.1371/journal.pone.0064675 23741366 PMC3669430

[B56] Marino K. A. Filizola M. (2018). Investigating small-molecule ligand binding to G protein-coupled receptors with biased or unbiased molecular dynamics simulations, in Computational Methods for GPCR Drug Discovery, ed Heifetz A. (New York, NY:Humana Press), 351–364. 10.1007/978-1-4939-7465-8_17 PMC5745006 29188572

[B57] Melo F. Sanchez R. Sali A. (2002). Statistical potentials for fold assessment. Protein Sci. 11, 430–448. 10.1002/pro.110430 11790853 PMC2373452

[B58] Meyerhof W. Batram C. Kuhn C. Brockhoff A. Chudoba E. Bufe B.. (2010). The molecular receptive ranges of human TAS2R bitter taste receptors. Chem. Senses 35, 157–170. 10.1093/chemse/bjp092 20022913

[B59] Miao Y. McCammon J. A. (2016). G-protein coupled receptors: advances in simulation and drug discovery. Curr. Op. Struct. Biol. 41, 83–89. 10.1016/j.sbi.2016.06.008 27344006 PMC5154880

[B60] Michino M. Abola E. Participants G. D. Brooks C. L. I. I. I. Dixon J. S. Moult J.. (2009). Community-wide assessment of GPCR structure modelling and ligand docking: GPCR Dock 2008. Nat. Rev. Drug Discov. 8, 455–463. 10.1038/nrd2877 19461661 PMC2728591

[B61] Miszta P. Pasznik P. Jakowiecki J. Sztyler A. Latek D. Filipek S. (2018). GPCRM: a homology modeling web service with triple membrane-fitted quality assessment of GPCR models. Nucleic Acids Res. 46, W387–W395. 10.1093/nar/gky429 29788177 PMC6030973

[B62] Munk C. Harpsoe K. Hauser A. S. Isberg V. Gloriam D. E. (2016b). Integrating structural and mutagenesis data to elucidate GPCR ligand binding. Curr. Opin. Pharmacol. 30, 51–58. 10.1016/j.coph.2016.07.003 27475047 PMC6910865

[B63] Munk C. Isberg V. Mordalski S. Harpsøe K. Rataj K. Hauser A. S.. (2016a). GPCRdb: the G protein-coupled receptor database–an introduction. Br. J. Pharmacol. 173, 2195–2207. 10.1111/bph.13509 27155948 PMC4919580

[B64] Munk C. Mutt E. Isberg V. Nikolajsen L. F. Bibbe J. M. Flock T.. (2019). An online resource for GPCR structure determination and analysis. Nat. Methods 16, 151–162. 10.1038/s41592-018-0302-x 30664776 PMC6881186

[B65] Musiani F. Giorgetti A. Carloni P. (2015). Molecular Mechanics/Coarse- grain simulations as a structural prediction tool for GPCRs/ligand complexes, in In Silico Drug Discovery and Design: Theory, Methods, Challenges and Applications, ed Cavasotto C. N. (Boca Raton, FL: CRC Press), 337–352.

[B66] Neri M. Anselmi C. Cascella M. Maritan A. Carloni P. (2005). Coarse- grained model of proteins incorporating atomistic detail of the active site. Phys. Rev. Lett. 95:218102. 10.1103/PhysRevLett.95.218102 16384187

[B67] Neri M. Baaden M. Carnevale V. Anselmi C. Maritan A. Carloni P. (2008). Microseconds dynamics simulations of the outer-membrane protease T. Biophys. J. 94, 71–78. 10.1529/biophysj.107.116301 17827219 PMC2134885

[B68] Nordstrom K. J. V. Almen M. S. Edstam M. M. Fredriksson R. Schioth H. B. (2011). Independent HHsearch, Needleman-Wunsch-based, and motif analyses reveal the overall hierarchy for most of the G protein-coupled receptor families. Mol. Biol. Evol. 28, 2471–2480. 10.1093/molbev/msr061 21402729

[B69] Olivella M. Gonzalez A. Pardo L. Deupi X. (2013). Relation between sequence and structure in membrane proteins. Bioinformatics 29, 1589–1592. 10.1093/bioinformatics/btt249 23677941

[B70] Oostenbrink C. Villa A. Mark A. E. Van Gunsteren W. F. (2004). A biomolecular force field based on the free enthalpy of hydration and solvation: the GROMOS force-field parameter sets 53A5 and 53A6. J. Comput. Chem. 25, 1656–1676. 10.1002/jcc.20090 15264259

[B71] Pándy-Szekeres G. Munk C. Tsonkov T. M. Mordalski S. Harpsøe K. Hauser A. S.. (2017). GPCRdb in 2018: adding GPCR structure models and ligands. Nucleic Acids Res. 46:D440–6. 10.1093/nar/gkx1109 29155946 PMC5753179

[B72] Pardo L. Deupi X. Dölker N. López-Rodríguez M. L. Campillo M. (2007). The role of internal water molecules in the structure and function of the rhodopsin family of G protein-coupled receptors. Chem. Bio. Chem. 8, 19–24. 10.1002/cbic.200600429 17173267

[B73] Piccoli S. Suku E. Garonzi M. Giorgetti A. (2013). Genome-wide membrane protein structure prediction. Curr. Genomics 14, 324–329. 10.2174/13892029113149990009 24403851 PMC3763683

[B74] Pydi S. P. Bhullar R. P. Chelikani P. (2014). Constitutive activity of bitter taste receptors (T2Rs). Adv. Pharmacol. 70, 303–326. 10.1016/B978-0-12-417197-8.00010-9 24931200

[B75] Pydi S. P. Jafurulla M. Wai L. Bhullar R. P. Chelikani P. Chattopadhyay A. (2016). Cholesterol modulates bitter taste receptor function. Biochim. Biophys. Acta 1858, 2081–2087. 10.1016/j.bbamem.2016.06.005 27288892

[B76] Ranganathan A. Rodríguez D. Carlsson J. (2017). Structure-based discovery of GPCR ligands from crystal structures and homology models, in Top Medicinal Chemistry (Berlin, Heidelberg: Springer).

[B77] Sandal M. Behrens M. Brockhoff A. Musiani F. Giorgetti A. Carloni P.. (2015). Evidence for a transient additional ligand binding site in the TAS2R46 bitter taste receptor. J. Chem. Theory Comput. 11, 4439–4449. 10.1021/acs.jctc.5b00472 26575934

[B78] Sandal M. Duy T. P. Cona M. Zung H. Carloni P. Musiani F.. (2013). GOMoDo: a GPCRs online modeling and docking webserver. PLoS ONE 8:e74092. 10.1371/journal.pone.0074092 24058518 PMC3772745

[B79] Schioth H. B. Fredriksson R. (2005). The GRAFS classification system of G-protein coupled receptors in comparative perspective. Gen. Comp. Endocrinol. 142, 94–101. 10.1016/j.ygcen.2004.12.018 15862553

[B80] Schneider J. Korshunova K. Musiani F. Alfonso-Prieto M. Giorgetti A. Carloni P. (2018). Predicting ligand binding poses for low-resolution membrane protein models: perspectives from multiscale simulations. Biochem. Biophys. Res. Commun. 498, 366–374. 10.1016/j.bbrc.2018.01.160 29409902

[B81] Schuler L. D. Daura X. Van Gunsteren W. F. (2001). An improved GROMOS96 force field for aliphatic hydrocarbons in the condensed phase. J. Comput. Chem. 22 1205–1218. 10.1002/jcc.1078

[B82] Schuler L. D. Van Gunsteren W. F. (2000). On the choice of dihedral angle potential energy functions for n-alkanes. Mol. Simulat. 25, 301–319. 10.1080/08927020008024504

[B83] Sengupta D. Joshi M. Athale C. A. Chattopadhyay A. (2016). What can simulations tell us about GPCRs: integrating the scales, in Methods in Cell Biology, ed Shukla A. K. (Cambridge, MA: Academic Press) 132, 429–452. 10.1016/bs.mcb.2015.11.007 26928554

[B84] Shaik F. A. Singh N. Arakawa M. Duan K. Bhullar R. P. Chelikani P. (2016). Bitter taste receptors: extraoral roles in pathophysiology. Int. J. Biochem. Cell Biol. 77(Pt B), 197–204. 10.1016/j.biocel.2016.03.011 27032752

[B85] Shen M. Y. Sali A. (2006). Statistical potential for assessment and prediction of protein structures. Protein Sci. 15, 2507–2524. 10.1110/ps.062416606 17075131 PMC2242414

[B86] Soding J. Biegert A. Lupas A. N. (2005). The HHpred interactive server for protein homology detection and structure prediction. Nucleic Acids Res. 33, W244–W248. 10.1093/nar/gki408 15980461 PMC1160169

[B87] Suku E. Fierro F. Giorgetti A. Alfonso-Prieto M. Carloni P. (2017). Multi- scale simulations of membrane proteins: the case of bitter taste receptors. J. Sci. Adv. Mat. Dev. 2, 15–21. 10.1016/j.jsamd.2017.03.001

[B88] Tarenzi T. Calandrini V. Potestio R. Carloni P. (2019). Open boundary-molecular mechanics / coarse grained framework for simulations of low-resolution G-protein-coupled receptor/ligand complexes. J. Chem. Theory Comput. 15, 2101–2109. 10.1021/acs.jctc.9b00040 30763087 PMC6433333

[B89] Tarenzi T. Calandrini V. Potestio R. Giorgetti A. Carloni P. (2017). Open boundary simulations of proteins and their hydration shells by Hamiltonian adaptive resolution scheme. J. Chem. Theory Comput. 13, 5647–5657. 10.1021/acs.jctc.7b00508 28992702

[B90] Tehan B. G. Bortolato A. Blaney F. E. Weir M. P. Mason J. S. (2014). Unifying family A GPCR theories of activation. Pharmacol. Ther. 143, 51–60. 10.1016/j.pharmthera.2014.02.004 24561131

[B91] Thal D. M. Glukhova A. Sexton P. M. Christopoulos A. (2018). Structural insights into G-protein-coupled receptor allostery. Nature 559, 45–53. 10.1038/s41586-018-0259-z 29973731

[B92] Tikhonova I. G. Fourmy D. (2010). The family of G protein-coupled receptors: an example of membrane proteins. Methods Mol. Biol. 654, 441–454. 10.1007/978-1-60761-762-4_23 20665280

[B93] Torrens-Fontanals M. Stepniewski T. M. Rodríguez-Espigares I. Selent J. (2018). Application of biomolecular simulations to G protein-coupled receptors (GPCRs), in Biomolecular Simulations in Structure-Based Drug Discovery, eds Gervasio F. L. Spiwok V. Mannhold R. Buschmann H. Holenz J. (Weinheim: Wiley-VCH), 205–223.

[B94] Trott O. Olson A. J. (2010). AutoDock Vina: improving the speed and accuracy of docking with a new scoring function, efficient optimization, and multithreading. J. Comput. Chem. 31, 455–461. 10.1002/jcc.21334 19499576 PMC3041641

[B95] Vanni S. Neri M. Tavernelli I. Rothlisberger U. (2011). Predicting novel binding modes of agonists to β adrenergic receptors using all-atom molecular dynamics simulations. PLoS Comput. Biol. 7:e1001053. 10.1371/journal.pcbi.1001053 21253557 PMC3017103

[B96] Velgy N. Hedger G. Biggin P. C. (2018). GPCRs: What can we learn from molecular dynamics simulations?, in Computational Methods for GPCR Drug Discovery. (New York, NY: Humana Press), 133–158. 10.1007/978-1-4939-7465-8_6 29188561

[B97] Venkatakrishnan A. J. Deupi X. Lebon G. Tate C. G. Schertler G. F. Babu M. M. (2013). Molecular signatures of G-protein-coupled receptors. Nature 494, 185–194. 10.1038/nature11896 23407534

[B98] Venkatakrishnan A. J. Ma A. K. Fonseca R. Latorraca N. R. Kelly B. Betz R. M.. (2019). Diverse GPCRs exhibit conserved water networks for stabilization and activation. Proc. Natl. Acad. Sci. U.S.A. 116, 3288–3293. 10.1073/pnas.1809251116 30728297 PMC6386714

[B99] Venter J. C. Adams M. D. Myers E. W. Li P. W. Mural R. J. Sutton G. G.. (2001). The sequence of the human genome. Science 291, 1304–1351. 10.1126/science.1058040 11181995

[B100] Webb B. Sali A. (2016). Comparative protein structure modeling using MODELLER. Curr. Protoc. Protein Sci. 86, 2.9.1–2.9.37. 10.1002/cpps.20 27801516

[B101] Worth C. L. Kreuchwig F. Tiemann J. K. Kreuchwig A. Ritschel M. Kleinau G.. (2017). GPCR-SSFE 2.0—a fragment-based molecular modeling web tool for Class A G-protein coupled receptors. Nucleic Acids Res. 45, W408–W415. 10.1093/nar/gkx399 28582569 PMC5570183

[B102] Xue A. Y. Di Pizio A. Levit A. Yarnitzky T. Penn O. Pupko T. (2018). Independent evolution of strychnine recognition by bitter taste receptor subtypes. Front. Mol. Biosci. 5:9 10.3389/fmolb.2018.00009 29552563 PMC5840161

[B103] Zhang J. Yang J. Jang R. Zhang Y. (2015). GPCR-I-TASSER: a hybrid approach to G protein-coupled receptor structure modeling and the application to the human genome. Structure 23, 1538–1549. 10.1016/j.str.2015.06.007 26190572 PMC4526412

[B104] Zhang Y. DeVries M. E. Skolnick J. (2006). Structure modeling of all identified G protein–coupled receptors in the human genome. PLoS Comput. Biol. 2:e13. 10.1371/journal.pcbi.0020013 16485037 PMC1364505

